# Alternative Materials for the Enrichment of Biogas with Methane

**DOI:** 10.3390/ma14247759

**Published:** 2021-12-15

**Authors:** Mieczysław Bałys, Ewelina Brodawka, Grzegorz Stefan Jodłowski, Jakub Szczurowski, Marta Wójcik

**Affiliations:** 1 Department of Coal Chemistry and Environmental Sciences, Faculty of Energy and Fuels, AGH University of Science and Technology, 30-059 Krakow, Poland; balys@agh.edu.pl (M.B.); szczurow@agh.edu.pl (J.S.); 2 Department of Fuels Technology, Faculty of Energy and Fuels, AGH University of Science and Technology, 30-059 Krakow, Poland; jodlowsk@agh.edu.pl (G.S.J.); mwojcik@agh.edu.pl (M.W.)

**Keywords:** adsorbents, carbonaceous materials, biogas, separation, biomethane

## Abstract

Carbonaceous adsorbents have been pointed out as promising adsorbents for the recovery of methane from its mixture with carbon dioxide, including biogas. This is because of the fact that CO_2_ is more strongly adsorbed and also diffuses faster compared to methane in these materials. Therefore, the present study aimed to test alternative carbonaceous materials for the gas separation process with the purpose of enriching biogas in biomethane and to compare them with the commercial one. Among them was coconut shell activated carbon (AC) as the adsorbent derived from bio-waste, rubber tire pyrolysis char (RPC) as a by-product of waste utilization technology, and carbon molecular sieve (CMS) as the commercial material. The breakthrough experiments were conducted using two mixtures, a methane-rich mixture (consisting of 75% CH_4_ and 25% CO_2_) and a carbon dioxide-rich mixture (containing 25% CH_4_ and 75% CO_2_). This investigation showed that the AC sample would be a better candidate material for the CH_4_/CO_2_ separation using a fixed-bed adsorption column than the commercial CMS sample. It is worth mentioning that due to its poorly developed micropore structure, the RPC sample exhibited limited adsorption capacity for both compounds, particularly for CO_2_. However, it was observed that for the methane-rich mixture, it was possible to obtain an instantaneous concentration of around 93% CH_4_. This indicates that there is still much potential for the use of the RPC, but this raw material needs further treatment. The Yoon–Nelson model was used to predict breakthrough curves for the experimental data. The results show that the data for the AC were best fitted with this model.

## 1. Introduction

The development of fuel production based on renewable resources is one the major trends in science and the economy. Climate change is an undeniable fact and a novel approach to acquiring useful fuels is a necessary step in civilization and social growth. Enriching biogas in methane will bring us closer to the use of renewable gas fuels in many branches, e.g., heat and power production [1,2,3], automotive propelling [4,5], and fuel cell energy production [6,7,8]. Different strategies are applied in order to transform biogas into useful fuels [9]. Current technologies allow for much more efficient removal of methane from its gas mixtures [10]. These methods are mostly based on physical or physicochemical phenomena [10,11,12] such as adsorption [13], absorption [14], membrane separation [15], or cryogenic techniques [16]. Among them, there exist also other ones, for example, chemical catalytic transformation [17,18,19], microbial electrochemical technologies [20], or hydrate-based processes [21].

In this paper we focus on adsorption technologies, especially materials (in this case, alternative ones), for which proper selection is a key factor in the successful use of this technology in biogas upgrading and cleaning [22]. Typical adsorbents used for the adsorptive separation of methane from carbon dioxide are usually carbon materials [23], such as activated carbons [24,25], carbon molecular sieves [26], and the increasingly popular MOFs [27,28], as well as their modified forms [29]. As waste materials, chars obtained from the pyrolysis of rubber tires have great potential [30], the same as adsorbents produced from biomass [31,32,33]. In addition, these chars were reported to exhibit reasonable adsorption properties for the cleaning of wastewater of iron [34], drugs [35], and dyes [36]. Although commercial carbon materials can be developed especially for the process of methane separation from the CH_4_/CO_2_ mixture (or with other components such as NH_3_, H_2_S, and N_2_), the cost of such an adsorbent is high in comparison to by-products of utilization processes or waste products from food production. Both paths, utilization of chars from pyrolysis (e.g., rubber tires, waste plastics, precipitation from carbonaceous materials gasification) or treatment of food production residues (e.g., coconuts, walnuts, fruit peelings, bones) are an eco-friendly way to management the waste products in the spirit of the circular economy [37,38,39]. Furthermore, one of the trends in science is the investigation of low-cost alternative adsorbents for different purposes [40], such as water cleaning from heavy metals [41,42], pharmaceuticals [43] or dyes [44]. Some applications of alternative adsorbents are targeted for gas purification, e.g., from mercury [45] and carbon dioxide capture [46], and also, eventually, for biogas purification [47,48]. Nevertheless, it is difficult to find publications on the use of waste tire pyrolysis char for gas separation. In addition, natural organic materials cannot be directly applied for gas separation without treatment, such as carbonization and/or activation, or complete restructuration of the material, including alkali activation [49,50] or metal activation [51]. From that point of view, they are costly due to the need for technological treatment, but they are surely an eco-friendly and renewable resource of “raw” carbonaceous materials. A different situation occurs when chars from waste materials pyrolysis are applied. These materials may characterize certain adsorption properties, so they could be used without further treatment, which will definitely influence the cost limitation. Thus, it is important to investigate their usefulness for the specified type of gas separation process.

Taking into consideration the adsorption-based process, especially pressure swing adsorption (PSA), as a method to enrich methane from biogas [52], a useful tool to check the viability of such a separation is the performance of a breakthrough curve experiment [12,27]. In this way, it is possible to evaluate the performance of potential adsorbent materials and their use for upgrading biogas for transport applications or to satisfy pipeline specifications, naturally, without complicated full adsorption–desorption PSA cycles. A large amount of research has been devoted to study the single-component adsorption of CO_2_ and CH_4_ in carbons and other materials. There is a scarcity of papers concerning multicomponent separation; moreover, most of them describe simulations of this separation, not experimental results. Furthermore, breakthrough curves of the mixture of CH_4_/CO_2_ for alternative materials such as chars obtained from the pyrolysis of rubber tires are lacking.

In this work, an evaluation of alternative carbonaceous materials for the gas separation process with the purpose of biogas enriching in biomethane is presented and discussed. The breakthrough experiments were conducted using two mixtures. Knowing that biogas has differing compositions of individual components that depends on the biogas source, we chose two opposite mixtures—a methane-rich mixture (containing 75% CH_4_ and 25% CO_2_) and a carbon dioxide-rich mixture (containing 25%CH_4_ and 75% CO_2_). Therefore, the main objective was to analyze the effect of the extreme contents of CH_4_ and CO_2_ in the feed stream and, based on these results, to prove that these materials are suitable for biogas separation.

## 2. Materials

Three types of carbonaceous materials were examined: carbon molecular sieve (CMS, Carbo-Tech) as a commercial material, coconut shell activated carbon (AC, Cocarb Solution) as an adsorbent derived from bio-waste, and rubber tire pyrolysis char (RPC, ReOil) as a by-product of waste utilization technology. The first two materials are activated carbons prepared for adsorption processes, and the RPC is a raw by-product not processed to increase sorption properties. The point of view of the authors is to find cheap materials for the technological process.

**Figure 1:**
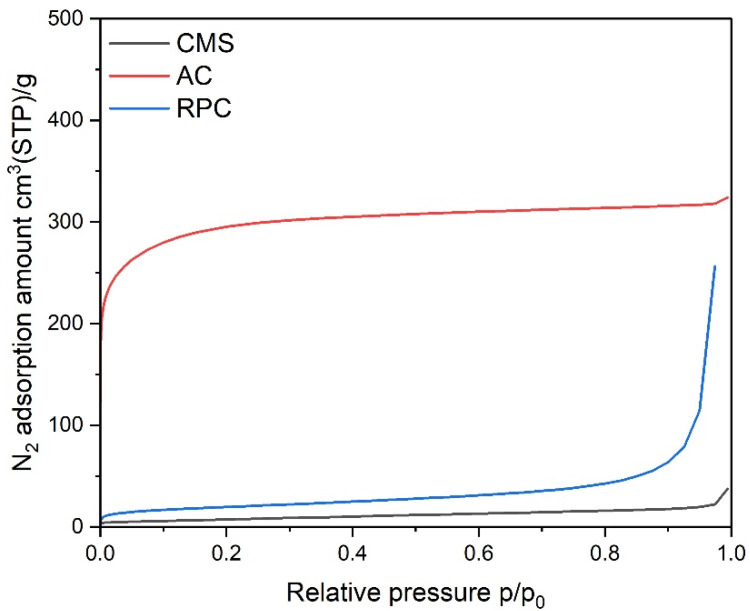
Isotherms of N_2_ adsorption on the investigated samples.

The materials were initially characterized by measurements of nitrogen adsorption isotherms, and data were analyzed according to the BET, D-R, and BJH theories of adsorption. Samples of the adsorbents were degassed under a deep vacuum for 12 h at 473 K. The N_2_ adsorption isotherms (see Figure 1) were measured at 77 K in the Autosorb 1-C (Qunatchrome) volumetric apparatus.

**Figure 2:**
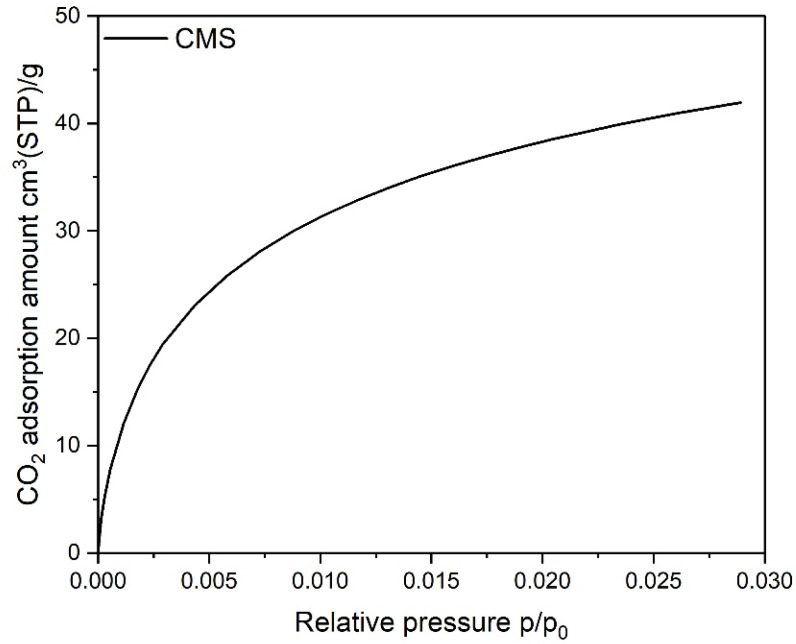
Isotherms of CO_2_ adsorption on the CMS sample.

It is important to realize that for the CMS, diffusion of nitrogen molecules at this temperature was kinetically hindered and, consequently, slower. Because of that, the isotherm measured was under-equilibrated and was not satisfactory with regard to a quantitative assessment of the microporosity, especially in the range of ultramicropores (pore widths < 0.7 nm). Consequently, a carbon dioxide adsorption isotherm at 273 K was measured for this CMS (see Figure 2) and data were analyzed according to the D-R method. The BET specific surface area and the pore volumes calculated from the N_2_ isotherm for the AC and RPC, as well as the micropore volume calculated from the CO_2_ isotherm for the CMS, are listed in Table 1. The calculated value of the BET surface area is significantly higher for the AC than the RPC.

The measured adsorption isotherms of N_2_ and CO_2_ are presented in Figure 1 and Figure 2, respectively. The AC material shows a type I isotherm with a narrow knee at low relative pressure, meaning that mainly micropores are present in this material. The RPC material is exhibited as an isotherm of type II, which is characteristic of low porous materials.

## 3. Experiment

**Figure 3:**
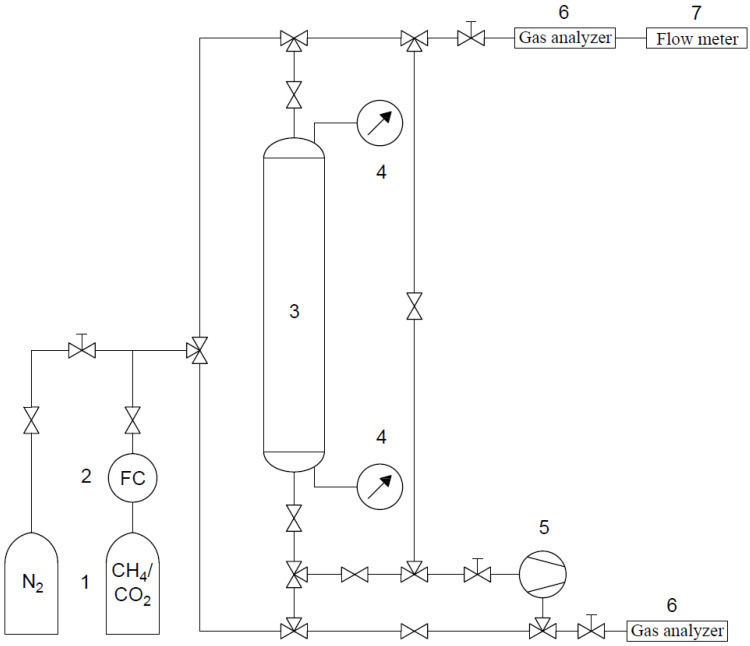
Scheme of the breakthrough apparatus: 1—gas bottles; 2—mass flow controller; 3 adsorption column; 4—pressure sensors; 5—vacuum pump; 6—gas analyzers; 7—flow meter.

The experimental apparatus for the breakthrough measurements, schematically shown in Figure 3, consisted of a column with a length of 1.5 m and an internal diameter of 0.02 m and packed with an adsorbent. For clarity, the necessary components are marked on Figure 3 with the valves omitted. It is worth pointing out that a high sample mass (249.15 g of CMS, 184.89 g of AC, and 151.65 g of RPC) was required to conduct the experiments. It was derived from targeting similar, close-to-real conditions in all of the experiments by means of the long column. A gaseous mixture of a bottle of known concentration was fed into the column. Two mixtures were studied, the first consisting of 75% CH_4_/25% CO_2_ and the second of 25% CH_4_/75% CO_2_. The measurements were carried out at atmospheric pressure and, depending on the sample, the feed flow was adjusted between 6 and 10 mL/s. The pressures at the bottom and top of the column were measured by pressure transmitters. Before the first and after the completion of other measurements, complete bed regeneration was carried out by a continuous flow of N_2_ until the exit concentration of CO_2_ and CH_4_ was equal to or close to zero. A vacuum pump was used to decrease the pressure below atmospheric during regeneration. The effluent stream was analyzed using a suitable detector to monitor and record data of the adsorbate breakthrough. Due to the use of pneumatic tubing and pneumatic connectors, flexible and easy manipulation of the apparatus was possible, thereby allowing gas flow in both directions. Thus, for the determination of the breakthrough curve, the feed flow was cocurrent, while in the regeneration step the nitrogen flow was carried out in both directions (cocurrent and countercurrent).

## 4. Column Dynamics Study

The dynamic behavior of adsorption in a fixed-bed column has been presented very often by breakthrough curves [53]. One of the most common methods used to accurately describe breakthrough curves is to use simple models without numerical solutions. There are several models that describe S-shaped breakthrough curves; among these, the most useful are the Thomas model, the Bohart–Adams model, and the Yoon–Nelson model [53]. In this study, the last one mentioned was applied to investigate the breakthrough behavior of CO_2_ on the selected carbons. This model, which does not include the properties of an adsorbate, type of adsorbent and any physical features of an adsorption bed, assumes that the probability of decreasing the rate of adsorption of each adsorbate molecule is directly proportional to the probability of the adsorbate adsorption and breakthrough on the adsorbent. The Yoon–Nelson model for a single component system [54] is expressed as:

(1) $$\frac{C}{C_{0}-C}=\exp(K_{YN}t-K_{YN}\tau_{1/2,YN})$$

where $K_{YN}$ is the rate constant of Yoon–Nelson and $\tau_{1/2}$ is the time required for retaining 50% of the initial adsorbate. The values of $\tau_{1/2,YN}$ and $K_{YN}$ in the Yoon–Nelson equation were determined by non-linear regression analysis.

Based on the Yoon–Nelson model, the amount of adsorbate being adsorbed in a fix-bed is half of the total adsorbate entering adsorption bed within the τ period [55]. Thus, the theoretical dynamic adsorption capacity of a column, $q_{YN}$ (mmol/g) is given as in the equation:

(2) $$q_{YN}=\frac{QC_{0}\tau_{1/2,YN}}{m}$$

Here, $C_{0}$ is the concentration of CO_2_ in the feed stream, Q is the feed molar flow rate, and m is the mass of the adsorbent in the bed.

To compare the theoretical dynamic adsorption capacity of a column $q_{YN}$ obtained using the Yoon–Nelson parameter, the area above the breakthrough curve at the column outlet was estimated by numerical integration for each experimental breakthrough curve, according to the following equation [56]:

(3) $$t_{st}=\overset{\infty }{\underset{0}{\int }}\left(1-\frac{C}{C_{0}}\right)dt$$

where $t_{st}$ is the stoichiometric time and C=C(t) is the concentration of CO_2_ in the outlet stream.

Then, using Equation (2) the experimental dynamic adsorption capacity of a column $q_{exp}\;$was calculated.

The ability of the Yoon–Nelson model to describe carbon dioxide adsorption was assessed using the coefficient of determination R^2^. The root mean square error (RMSE) was also calculated.

## 5. Results and Discussion

The values of $K_{YN}\;$and $\tau_{1/2}$ are listed in Table 2. For all of the samples, the rate constant $K_{YN}$ increased and the $\tau_{1/2,YN\;}$ decreased with decreasing contents of CH_4_ in the mixtures analyzed.

**Figure 4:**
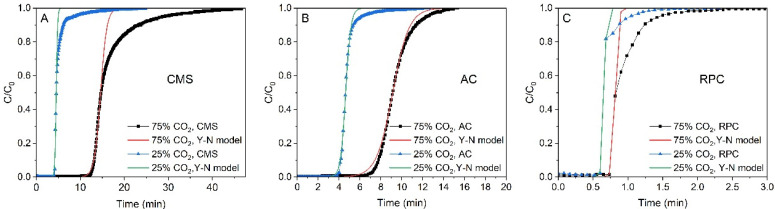
The experimental and predicted breakthrough curves of CO_2_ for CMS (**A**), AC (**B**), and RPC (**C**) samples at 1 bar and 293 K.

The experimental breakthrough curves are compared to the predicted ones in Figure 4. The data for the AC were best fitted with the Yoon–Nelson model. For the CMS and RPC samples the predicted breakthrough curves were in good agreement with the experimental data in the range of (C/C_0_) > 0.5 and departed for (C/C_0_) < 0. This is because of the fact that for the AC sample the breakthrough curves were symmetrical and S-shaped, while for the CMS, the curves were asymmetrical. As mentioned above, the Yoon–Nelson model fits the experimental data to the model data without much error only for the symmetrical curves.

**Figure 5:**
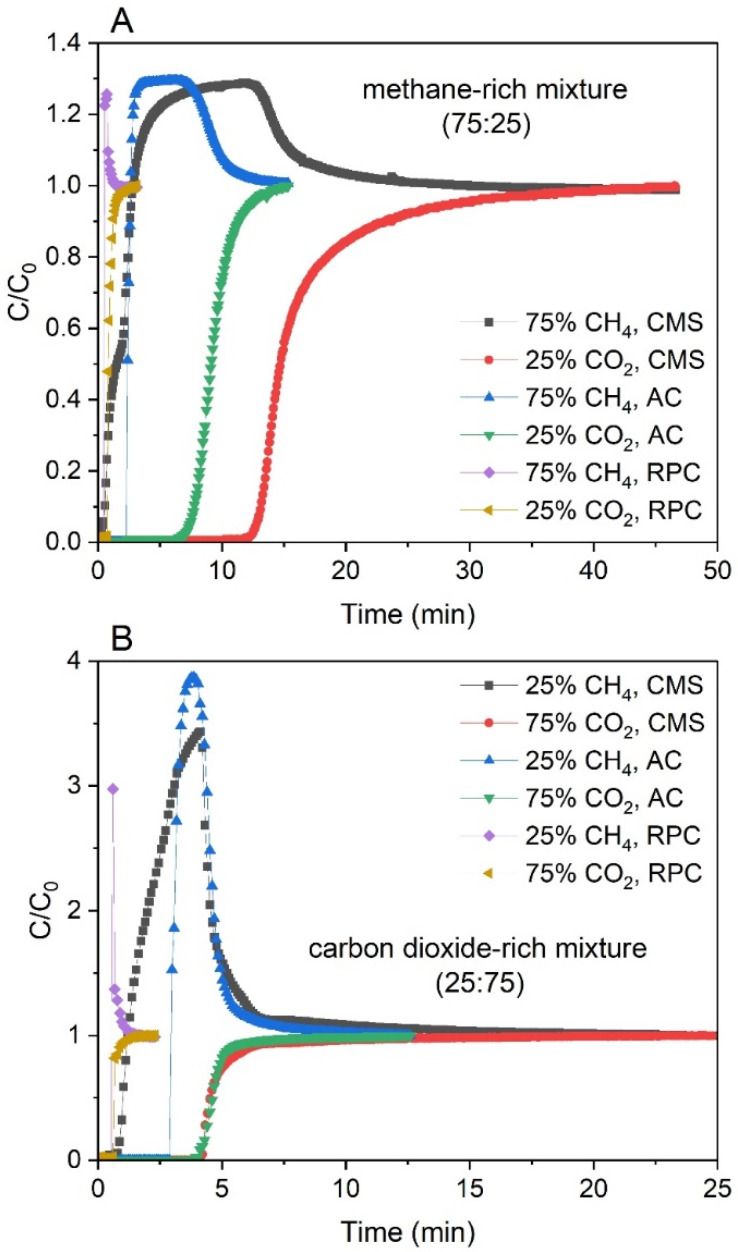
Breakthrough curves of CH_4_ and CO_2_ collected for all of the samples for the methane-rich mixture (**A**) and the carbon dioxide-rich mixture (**B**) at 1 bar and 293 K.

For all the samples, if the predicted curves were fitted to the points below $\tau_{1/2}$ then the Yoon–Nelson model described the beginning of the process well, whereas above the $\tau_{1/2}\;$point the goodness of fit decreased (especially for the CMS). For such a fitted curve, the calculated $K_{YN}$ value was overestimated in that region. Therefore, the reliability of the $K_{YN}$ value for the CMS is questionable. In Figure 5 it was observed that the breakthrough curves obtained for the CMS were less steep compared with those obtained for the AC for both mixtures. Thus, the value of the rate constant $K_{YN}\;$for the AC sample should be greater than that for the CMS sample.

**Figure 6:**
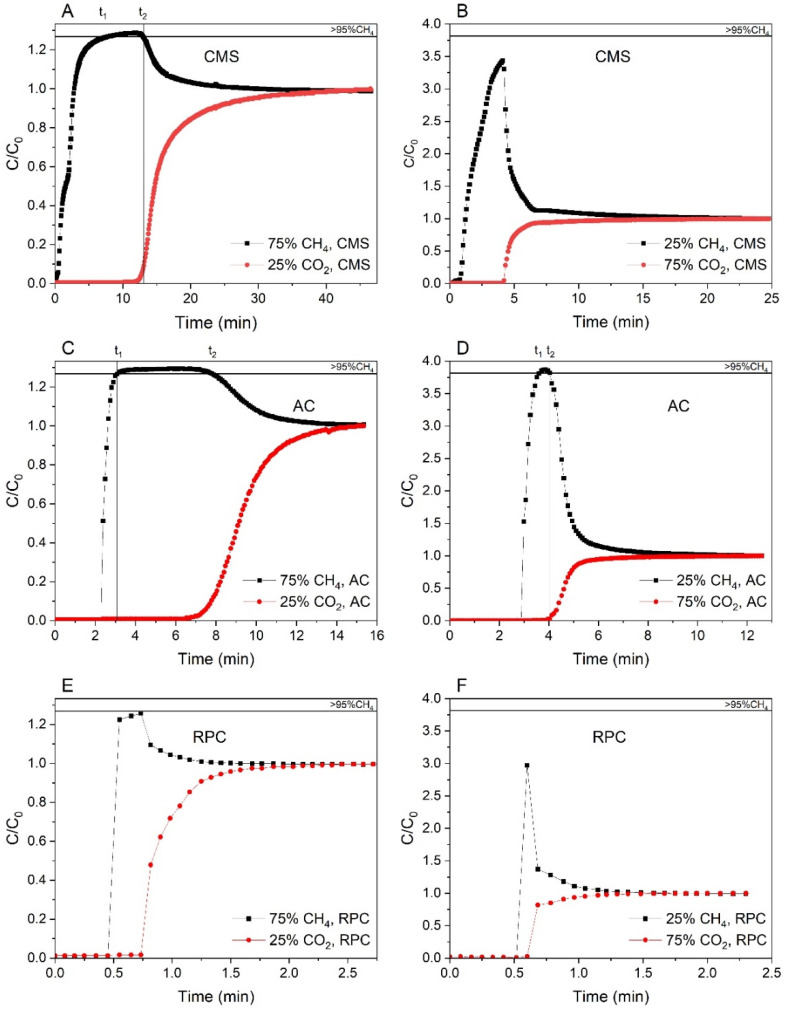
Breakthrough curves for the CMS (**A**,**B**), AC (**C**,**D**), and RPC (**E**,**F**) samples of the methane-rich mixture and the carbon dioxide-rich mixture, respectively, at 1 bar and 293 K.

The breakthrough curves of the methane-rich mixture and carbon dioxide-rich mixture at atmospheric pressure for the CMS, AC, and RPC samples are shown in Figure 6. The breakthrough curves are represented in the form of the normalized molar flow rates C/C_0_, C being the measured flow rate of the component at the column outlet and C_0_ being the feed flow rate of the component. It was observed that CH_4_ always broke first, and its breakthrough curve exhibited a so-called roll-up. This effect can be explained as follows: CH_4_ as the less adsorbed component loses its adsorption sites due to the competitive adsorption of the more strongly adsorbed component (in that case carbon dioxide), which is retained within the pores. This results in a higher concentration of CH_4_, which can be higher than its feed concentration. Moreover, with increasing composition of the CH_4_, the roll-up of this component becomes progressively less pronounced [57,58]. It could also be observed in Figure 6.

It should be noted that the integration of the area above C/C_0_ = 1 of the CH_4_ breakthrough curve (called the roll-up) is counted as a negative adsorbed amount [57]. Due to this fact, the molar productivity of CH_4_ with the selected purity level was calculated by integrating the CH_4_ molar flow rate profile into the outlet gas between the time interval of *t_1_* to *t_2_* (where CH_4_ can be produced with the purity selected, in our case above 95%) as follows [58]:

(4) $$mol.prod.{\mathrm{CH}}_{4}=\frac{1}{m}\int_{t_{1}}^{t_{2}}F_{CH_{4},exit}dt$$

where $F_{CH_{4},exit}$ is the molar flow rate of CH_4_ that exits the bed and m is the mass of adsorbent packed in the bed. The molar productivity, as estimated from Equation (4), is then reported in moles per kilogram of adsorbent and is listed in Table 3 Additionally, in Table 3 the comparison between the theoretical and experimental dynamic adsorption capacities of a column is shown.

The calculated values of the experimental and theoretical dynamic adsorption capacities for all samples indicated that, as expected, these amounts increased with increasing CO_2_ content. For instance, the capacity values obtained for the AC sample rose from 0.195 to 0.335 mol/g adsorbent as the content increased from 25% in the methane-rich mixture to 75% in the carbon dioxide-rich mixture. This increase is likely related to the equilibrium isotherm point at which the bed is operating at any given time, that is, the higher the CO_2_ composition, the higher the partial pressure of CO_2_. Previous researchers have reported similar observations [59]. Based on the predicted adsorption isotherms of the binary gas mixture between CO_2_ and CH_4_ on zeolite 4A, it was shown that the higher the composition of CO_2_, the greater the amount of total gas adsorption.

Moreover, from Table 3 as well as from Figure 6, it can be seen that for the RPC the adsorption capacity is small compared to the rest of the materials. This is due to a poorly developed micropore structure that limits the adsorption of the mixture’s components. However, it was observed that for the methane-rich mixture, an instantaneous concentration of around 93% CH_4_ was obtained. This indicates that there is still much potential for the use of the RPC, but this raw material needs further treatment.

A further analysis of Table 3 showed that for the CMS sample, the adsorption capacities calculated according to Equation (2) were higher than for the AC for the methane-rich mixture. The opposite situation was observed for the carbon dioxide-rich mixture. These results suggested that the CMS has a general higher adsorption capacity related to CO_2_ than the AC, which was manifested in the case of the methane-rich mixture. For the carbon dioxide-rich mixture, this large amount of CO_2_ probably could not keep up with the adsorption on the CMS sample. Thus, the calculated adsorption capacity was lower than for the AC sample and, moreover, in Figure 5B a similar breakthrough time of the CO_2_ breakthrough curves can be observed for both samples. This can be attributed to a more complex mass transfer process, limited by slower methane adsorption and desorption for the CMS.

The last parameter to discuss in Table 3 was the molar productivity of CH_4_. It is worth mentioning that for the CMS sample, the maximum instantaneous concentration was 97.2% for the methane-rich mixture and 80.7% for the carbon-dioxide-rich mixture; for the AC these values were 98% and 96.4% and for the RPC 93.4% and 67.5%, respectively. Therefore, for the first mixture examined, the CH_4_ molar productivity could be calculated for all samples, but only for the AC this value was determined for the carbon dioxide mixture, because only in that case the concentration of CH_4_ reached the selected purity, which was 95 vol.%. It seems clear that according to the parameter of CH_4_ molar productivity, the AC sample would be a better candidate material for the CH_4_/CO_2_ separation with the purpose of enriching mixtures in methane than the remaining two.

## 6. Conclusions

In this work we compared three carbon materials, including two waste materials, for potential CH_4_/CO_2_ separation. In order to determine the porous texture of the carbon materials selected, the adsorption isotherms of nitrogen gas at 77 K were measured. The data were analyzed according to the BET, D-R, and BJH theories of adsorption. In addition, a breakthrough curve experiment was used to determine the performance of the materials under dynamic conditions. This investigation showed that the AC sample would be a better candidate material for the separation of CH_4_/CO_2_ using a fixed-bed adsorption column than the commercial CMS sample. Only for the AC, the purity of CH_4_ in the outlet stream for both mixtures examined was above 95 vol%. It should be mentioned that due to its poorly developed micropore structure, the RPC sample exhibited limited adsorption capacity for both compounds, particularly for CO_2_. However, it was observed that when the mixture contained 75% CH_4_, an instantaneous concentration of around 93% CH_4_ was obtained for that sample. This indicates that there is still much potential for the use of the RPC, but this raw material needs further treatment with the aim of increasing porosity, but we should be aware that it probably increases the cost of the material.

## Figures and Tables

**Table 1:** Characterization of the investigated samples.

Parameter	Unit	CMS ^a^	AC ^b^	RPC ^b^
S_BET_	m^2^/g	-	1118	70.3
V_mic_	cm^3^(STP)/g	0.177	0.415	0.025
V_mes_	cm^3^(STP)/g	-	0.061	0.390

^a^ Parameters for this adsorbent determined from CO_2_ isotherm; ^b^ parameters for this adsorbent determined from N_2_ isotherm.

**Table 2:** Yoon–Nelson model parameters and the stoichiometric time for the investigated samples.

Parameter	Unit	CMS		AC		RPC	
		75:25 ^a^	25:75 ^b^	75:25 ^a^	25:75 ^b^	75:25 ^a^	25:75 ^b^
$K_{YN}$	1/min	1.41	6.50	1.28	4.09	47.35	59.78
$\tau_{1/2,YN}$	min	14.60	4.53	9.13	4.64	0.82	0.66
$t_{st}$	min	16.69	5.14	9.29	4.75	0.89	0.65
R^2^	-	0.984	0.989	0.997	0.998	0.951	0.992
RMSE	-	0.053	0.038	0.023	0.023	0.090	0.039

^a^ Parameters for the methane-rich mixture (containing 75% CH_4_ and 25% CO_2_); ^b^ parameters for the carbon dioxide-rich mixture (containing 25% CH_4_ and 75% CO_2_).

**Table 3:** Theoretical and experimental dynamic adsorption capacities of a column and molar productivities of CH_4_.

Parameter	Unit	CMS		AC		RPC	
		75:25 ^a^	25:75 ^b^	75:25 ^a^	25:75 ^b^	75:25 ^a^	25:75 ^b^
$q_{YN}$	mmol/g	0.252	0.273	0.195	0.335	0.024	0.059
$q_{exp}$	mmol/g	0.286	0.309	0.198	0.344	0.026	0.059
$mol.prod.{\mathrm{CH}}_{4}$	mmol/g	0.433	- ^c^	0.467	0.033	- ^c^	- ^c^

^a^ Parameters for the methane-rich mixture (containing 75% CH_4_ and 25% CO_2_); ^b^ parameters for the carbon-dioxide-rich mixture (containing 25% CH_4_ and 75%CO_2_); ^c^ maximum concentration below the selected purity (95% CH_4_).

## Data Availability

Not applicable.
